# Multiple migrations from East Asia led to linguistic transformation in NorthEast India and mainland Southeast Asia

**DOI:** 10.3389/fgene.2022.1023870

**Published:** 2022-10-11

**Authors:** Debashree Tagore, Partha P. Majumder, Anupam Chatterjee, Analabha Basu

**Affiliations:** ^1^ National Institute of Biomedical Genomics, Kalyani, India; ^2^ Indian Statistical Institute, Kolkata, India; ^3^ Department of Biotechnology, North-Eastern Hill University, Shillong, India; ^4^ School of Biosciences, Royal Global University, Guwahati, India

**Keywords:** Austroasiatic, Khasi, Tibeto Burman, admixture, migration, linguistic transformation

## Abstract

NorthEast India, with its unique geographic location in the midst of the Himalayas and Bay of Bengal, has served as a passage for the movement of modern humans across the Indian subcontinent and East/Southeast Asia. In this study we look into the population genetics of a unique population called the Khasi, speaking a language (also known as the Khasi language) belonging to the Austroasiatic language family and residing amidst the Tibeto-Burman speakers as an isolated population. The Khasi language belongs to one of the three major broad classifications or phyla of the Austroasiatic language and the speakers of the three sub-groups are separated from each other by large geographical distances. The Khasi speakers are separated from their nearest Austroasiatic language-speaking sub-groups: the “Mundari” sub-family from East and peninsular India and the “Mon-Khmers” in Mainland Southeast Asia. We found the Khasi population to be genetically distinct from other Austroasiatic speakers, i.e. Mundaris and Mon-Khmers, but relatively similar to the geographically proximal Tibeto Burmans. The possible reasons for this genetic-linguistic discordance lie in the admixture history of different migration events that originated from East Asia and proceeded possibly towards Southeast Asia. We found at least two distinct migration events from East Asia. While the ancestors of today’s Tibeto-Burman speakers were affected by both, the ancestors of Khasis were insulated from the second migration event. Correlating the linguistic similarity of Tibeto-Burman and Sino-Tibetan languages of today’s East Asians, we infer that the second wave of migration resulted in a linguistic transition while the Khasis could preserve their linguistic identity.

## Introduction

The Indian subcontinent is genetically one of the most diverse regions of the world harboring over 1.25 billion people (2011 census). The region has been part of the earliest waves of Anatomically Modern Human (AMH) migrations which peopled South and Southeast Asia, including Australia, beginning around 60,000 years ago (Basu et al., 2003; Kivisild et al., 2003; Macaulay et al., 2005; Thangaraj et al., 2005). Over time, the region has also witnessed multiple waves of migration (Basu et al., 2003; Endicott, Metspalu, and Kivisild 2007; Majumder 2008; Basu et al., 2016) that has contributed to its huge genetic, linguistic, and cultural diversity. The Indian subcontinent is bounded in the North and Northeast by the Himalayas. NorthEast India (NEI) is a unique region that is bordered in the north by the high ranges of eastern Himalayas and two-thirds of it is intermediate hilly terrain, interspersed by fertile riverbeds and flat valleys. The population density also varies accordingly; while the river valleys are densely populated and cosmopolitan, the highlands are sparsely populated by small isolated ethno-lingual groups. Major population groups that reside here speak Tibeto-Burman languages, which belong to the non-Sinitic phylum of the Sino-Tibetan language family. Now restricted by political boundaries, this region is likely to have been a land bridge between peninsular India (PI) and Mainland Southeast Asia (MSEA) and has been an active corridor of migration and admixture of different ethnolinguistic populations in the past (Gadgil et al., 1993; Cavalli-Sforza et al., 1994; Reddy et al., 2007; Tagore et al., 2021; Liu et al., 2022) and hence should be considered in continuum with the population demographic history of East and Southeast Asia. Individuals from mainly five language families reside in NEI and the neighborhood of MSEA: namely Sino-Tibetan, Tai-Kadai, Hmong-Mein, Austronesian, and Austroasiatic (AA). However, more recent migrations of the ancestors of Indo-European language speakers of India, who possibly entered India through the northwestern corridor also had a large impact on the populations of NEI (Gayden et al., 2009; Basu et al., 2016). The Austroasiatic language family comprises three major subfamilies: Munda, Mon-Khmer, and Khasi-Khmuic (Diffloth Gerard 2005a). Within the Austroasiatic family, the Khasi language (the sole language of the Khasi-Khmuic branch of the Austroasiatic language family in India) is spoken in NEI mainly in parts of the north-eastern state of Meghalaya.

In this study we look into the population genetics of the Khasi, residing amidst the Tibeto-Burman speakers as an isolated population. These Khasi speakers are separated by large physical distance from their nearest Austroasiatic language-speaking sub-groups: the Munda sub-family from East and peninsular India and the Mon-Khmer sub-family in Mainland Southeast Asia. Here, we dissect the genetic relationship of the Khasis with the other Austroasiatic subgroups and in an attempt to do so, reconstruct the population history of NorthEast India, and the neighboring East and Southeast Asia, in the context of the Khasi Austroasiatics.

Despite a strategic location, most genetic studies on NEI populations have been done using either uniparental markers (Cordaux et al., 2003; Borkar et al., 2011) or a small number of autosomal markers (Maity, Nunga, and Kashyap 2003; Krithika et al., 2005; Krithika et al., 2006; Mastana et al., 2007; Gayden et al., 2009). Cordaux et al. (2003) mitochondrial DNA (mtDNA) and Y chromosome-based study suggest two possibilities regarding the peopling of NEI: either TBs were the earliest inhabitants, or the TB replaced the Austroasiatic (AA) inhabitants of NEI. Using microsatellite data, and comparing the Khasi-Khmuic speakers with their neighboring Tibeto-Burman speakers, showed the populations to be extremely homogeneous (Langstieh et al., 2004) a fact further supported by later studies with mtDNA and Y-chromosome (Cordaux et al., 2004). Initially, researchers came up with opposing views on the origin of TB populations. One theory, based on Y-chromosome analyses, suggests that the TB ancestors originated in the upper and middle Yellow River basin (Su et al., 2000). Another theory suggests the Yangtze River as their ancestral source followed by the northward movement to the Yellow River basin (Van Driem 2005). In our previous study (Tagore et al., 2021) we also proposed a theory where we suggested that present-day Tibeto Burmans were likely Austroasiatics in the past, who were part of the earliest settlers of the region (Hill et al., 2006). Y-chromosome based study by Wang et al. (2018) suggested that the peopling of the Tibetan plateau by Tibeto Burman ancestors happened some 40KYA (40 thousand years ago). This coincides with the presence of hunter gatherers in this region. However, it was during the Neolithic period, ∼6KYA, when the expansion of different Y chromosome lineages was observed leading to the present day distribution of the TBs. This time coincides with the migration of East Asians in MSEA (Tagore et al., 2021). Yu et al. (2021) have suggested that migration of both Yellow river basin millet farmers and Yangtze river basin rice farmers contributed to different linguistic and genetic groups in MSEA. They have also proposed that around 6KYA, people from the middle Yellow River Basin migrated south-westward and mixed with the local population to give rise to the initial TBs. Basu et al. (2003) has shown that the TB and AA speakers of India are similar in their mtDNA profile but harbor very distinct Y-chromosomes. Our previous study (Tagore et al., 2021) observed the genetic relatedness between the Tibeto Burmans and Austroasiatic speakers (Mon Khmers) of Malaysia, because of ancient shared ancestry as well as owing to gene flow in both these populations from East Asia. Another study (Guo et al., 2022) found the present day TBs to cluster between the millet cultivators of Yellow River basin as well as the Austroasiatic speakers of Southeast Asia in a Principal Components Analysis. In further analyses they found southern Tibeto Burmans were genetically closest to the AAs.

The Northeast Indian populations were clustered with populations of East and Southeast Asia than with mainland Indians (Langstieh et al., 2004; Basu et al., 2016; Tagore et al., 2021). Other studies on the mtDNA hypervariable region and autosomal microsatellite markers found that despite the present political boundary, the Tibeto-Burman speakers from NEI showed a closer genetic affinity with East Asian populations than with other mainland Indian populations (Cordaux et al., 2003; Krithika, Maji, and Vasulu 2008; Basu et al., 2016). This further supports the fact that ancient migration events occurred through the NEI corridor before the political boundaries were drawn (Basu et al., 2003; Basu et al., 2016). Such genetic studies are in agreement with the linguistics of Northeast India: the Tibeto Burman language group is closely related to the languages of East Asia. Apart from the genetic similarity with the East Asians, the TB also shows some complex admixture with other Indian populations belonging to different ancestries. The TBs harbor genetic ancestry predominantly in Indo-European speakers (henceforth referred to as ANI or Ancestral North Indian) who mainly reside in the northern part of India, and also genetic ancestry predominantly in Dravidian language speakers (henceforth referred to as ASI or Ancestral South Indian) who are almost exclusively confined to the southern part of India (Basu, Sarkar-Roy, and Majumder 2016).

The Khasis are one of the few populations in the world that follow a matrilineal system of inheritance. Besides the linguistic similarity, anthropologists and archaeologists have established that the Khasis have cultural similarities with Mundaris and Mon-Khmer populations. It has been shown that they share similar stone tools and have similar death rituals of erecting memorial stones for the deceased (Gurdon 1914). Linguistically, the Khasi language is more similar to languages of the Mon-Khmer branch than those of the Mundari branch and linguists have often assigned Khasi and Mon-Khmer languages to the same group (Pinnow et al., 1942; Chazée 1999). Khasi language also bears lexical and morphological similarities to some Tibeto Burman languages (Longmailai 2015). Peiros suggested a significant number of words were similar between Proto Austroasiatics and Proto-Sino-Tibetans (Peiros 2011).

Nevertheless, the presence of ancient Austroasiatics (AA) speakers across NEI still remains a possibility. Our previous study (Tagore et al., 2021) on autosomal data of the Mundari and Mon-Khmer Austroasiatics indicated that in pre-Neolithic times, the ancestors of today’s Austroasiatic speakers had a widespread distribution possibly extending from Central India to Southeast Asia (SEA), further supported by Lipson et al. (2018). They were later in time fragmented and isolated to small pockets resulting in their present-day disjoint geographic distribution. What is intriguing is that given the widespread distribution of Austroasiatic speakers from Central India to SEA across NEI and the central location of the Khasis, it is possible that the Khasis will serve as a genetic link between the two Austroasiatic groups on either side of NEI.

There have been very few studies on the genetics of Khasi, so as to reach any plausible conclusions. One previous study on uniparental markers has proposed a genetic continuity between the Mundari Austroasiatics of Central India, Khasi-Khmuic, and Mon-Khmer (Reddy et al., 2007). Using multidimensional scaling of the pairwise F_ST_ distances calculated on Y-haplogroups of Austroasiatics and neighboring populations, they found the three Austroasiatic groups (Mundari, Khasi-Khmuic, and Mon-Khmer) to cluster together. They also found the Y haplogroup O-M95, restricted within the Austroasiatics and postulated to have originated in the Mundaris, is present in the Khasis at a frequency intermediate to that of Mundaris and Mon-Khmers. They suggested an initial presence of Austroasiatics in Central India with rapid migration to Southeast Asia *via* the Northeast corridor carrying the O-M95 haplogroup.

The cultural and linguistic similarities of the Khasis with other Austraoasiatic groups as mentioned earlier prompt us to investigate their genetic affinities. The geographic location of the Khasis also makes it imperative to investigate the impact of East Asian migrations on the genetic make-up of the Khasis.

## Materials and methods

### Dataset preparation and quality control

DNA samples from 22 individuals speaking the Khasi language were sequenced and merged with the genotype dataset of 1,451 individuals that were used in our previous study (Tagore et al., 2021) using PLINK(Shaun et al., 2007). The details of the datasets are provided in Supplementary Table S1A–C. Only biallelic loci were included in our analysis. We removed all monomorphic variants and SNPs with alleles A/T and G/C from our analysis. We also removed SNPs with missingness of more than 5% in the entire dataset, or SNPs that were missing in more than 25% of individuals in any of the 15 subpopulations (second column of Supplementary Table S1A). We also excluded SNPs that were out of Hardy Weinberg equilibrium (*p* < 10^−6^) in any of the 15 subpopulations. This combined dataset had 310110 SNPs.

### Principal components analysis

In order to understand the overall population structure and the genetic affinities of the individuals in our dataset, we performed Principal Components Analyses (PCAs) using the smartpca program of the EIGENSOFT package (Patterson, Price, and Reich 2006). We performed an initial PCA on all the mainland Indians (all Indian populations excluding those belonging to the “Island” group as in Supplementary Table S1A) and Malaysian populations. We considered the first two Principal Components (PCs) to visualize the data.

A second PCA was run on a subset of populations used in the first PCA. This subset was chosen based on linguistic similarity and geographic proximity to the Khasis. Thus, we included the Austroasiatics from Central India (AACI), Austroasiatics of Malaysia (AAM), Khasi, and Tibeto Burmans (TBs).

### TreeMix

In order to understand how populations were related to each other through a common ancestor and the impact of genetic drift, we built ancestry graphs using TreeMix (Pickrell et al., 2012) version 1.12. Such graphs were created with AACI, AAM, TB, and the Khasi populations using the Mbuti Pygmies from Africa as an outgroup.

### F_st_ estimates

Using PLINK (Shaun et al., 2007) version 1.9, the weighted F_st_ between each subpopulation of AACI, AAM, TB, and the Khasi was estimated. These values were rounded to the third decimal place.

### Outgroup *f3* statistics

Outgroup *f3* statistics measures the shared drift between two populations relative to an extremely diverged population outgroup. Using ADMIXTOOLS (v5.1) (Patterson et al., 2006), we calculated outgroup *f3* statistics of the form *f3* (Mbuti Pygmy; Khasi, Y) where Mbuti Pygmy was the outgroup. Y was AACI, AAM, and TB subpopulations.

### ADMIXTURE analysis

To infer the different ancestral components present in the admixed populations and the proportions of each such component in an individual’s genome, we performed unsupervised clustering as implemented in ADMIXTURE (Alexander, Novembre, and Lange 2009) (v1.3.0). We ran ADMIXTURE using all Indian populations (AACI, ANI, ASI, ATB, and Khasi), Malaysian populations (AAM and ANS), and all East Asians. We ran ADMIXTURE by sequentially increasing the number of clusters, which corresponds to the number of identified ancestries (*k*), in each run of the analysis on a given dataset. ADMIXTURE estimates the proportion of each of the *k* ancestries in the genome of each individual of the dataset and also computes a cross-validation error (CVE) for that particular run. Standard error was estimated for the ancestry proportion estimates at the minimum CVE using the moving block bootstrap approach as implemented in ADMIXTURE.

### Admixed segment length calculation

Dataset was phased using SHAPEIT v2.r790 (Delaneau, Marchini, and Zagury 2012). From the phased dataset we extracted the phased genomes for Khasi, Jamatia, Miazou (a Southern East Asian-like ancestry population), Yakut (a Northern East Asian-like ancestry population), and Kshatriya (an ANI-like ancestry population). This was followed by local ancestry estimation using RFMix (Maples et al., 2013) version 1.5.4, to identify regions of genomes of Khasi and a TB population (in this case Jamatia) corresponding to different ancestries. The different ancestries which we considered for the local ancestry estimation were inferred from the ADMIXTURE run where the CVE was minimum, i.e. at *k = 8*. Ancestries for which tract lengths were estimated in Khasi included: “Southern EA-like”, “Jehai-like”, “MahMeri-like”, “Birhor-like (AACI-like)” and “ANI-like”. In addition to these ancestries, tract lengths corresponding to “Northern EA-like” ancestry were also estimated for Jamatia. It is to be noted here that the Northern EA-like ancestry was practically absent in the Khasis. We plotted the cumulative distribution of these tract lengths to compare the sizes of these tract lengths corresponding to the different ancestries in both Khasi and Jamatia.

### Estimating admixture time

Gene flow events between genetically distinct populations create linkage disequilibrium between all loci that are highly differentiated between the two ancestral populations. Segments resulting from admixture follow an exponential distribution, where as a result of recombination, this linkage disequilibrium pattern declines exponentially over time and from which the number of generations since admixture can be estimated (Racimo et al., 2015). To date the ‘time since the last admixture event’ between different populations, we generated “co-ancestry curves” using MOSAIC (Salter-Townshend and Myers 2019) (v1.2). Here the closest surrogate populations were chosen as “donors”. Coancestry curves measure how often, in an admixed (“recipient”) population; a pair of haplotypes has been inherited from each respective donor population. Given a single admixture event, ancestry chunks inherited from each source, reduce in size because of recombination, resulting in an exponential decay of these coancestry curves. The time (in generations) since admixture is calculated from the rate of decay in the curves.

To detect 2-way admixture events in Jamatia and Khasi, we used Yakut as a surrogate donor of the “Northern EA-like” ancestry, Miazou for “Southern EA-like” ancestry and Birhor for “Austroasiatic-India or Mundari” ancestry. We chose Khasi and Jamatia as recipients (having ancestry from each of the source populations as a result of admixture) and estimated the time since the last admixture between the donors. We created co-ancestry curves for the surrogate donors (Miazou and Birhor) in both Khasi and Jamatia and another co-ancestry curve for donors Yakut and Birhor in Jamatia. The rate of decay in the curves was calculated which was equal to the number of generations since admixture took place.

## Results

**FIGURE 1:**
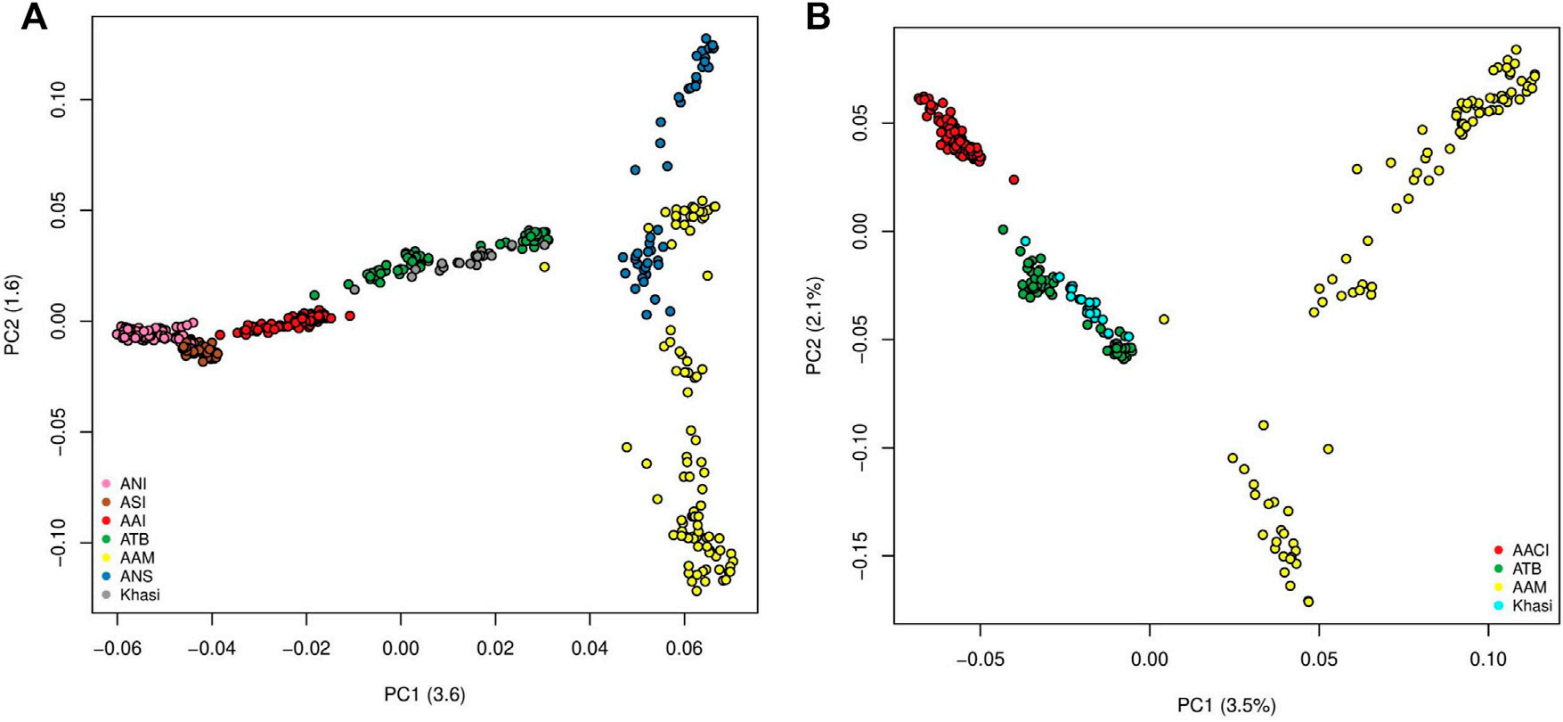
**(A)** PCA on Mainland Indian and Malaysian populations (ANI: Ancestral North Indian, ASI: Ancestral South Indian, AACI: Austroasiatics of Central India, ATB: Ancestral Tibeto Burmans, AAM: Austroasiatics of Malaysia, ANS: Austronesians); **(B)** PCA on the Austroasiatics and the Tibeto Burmans.

The first two Principal Components (PCs) of the PCA with all the mainland Indians (all Indian populations excluding those belonging to the “Island” group as in Supplementary Table S1A) and Malaysian populations explained 3.6% and 1.6% of the variation. In PC1-PC2 space the individuals belonging to the major population groups (as classified in the second column of Supplementary Table S1A), formed unique clusters. In the PC1 axis the Indian population, specifically the Ancestral North India-like (ANI-like) populations were on one extreme while the Malaysian populations were on the other. While most Indian populations were distinguishable along the first PC, the two Malaysian populations separated along the second PC. We found the Khasis to cluster with the Tibeto Burmans (Figure 1A) (It is to be noted that this is very similar to Figure 1D in Tagore et al., 2021 where we had all the populations except the Khasis). Using a smaller subset of the above we did a second PCA, where, we considered the Khasis along with the two Austroasiatic groups from our previous study i.e. Mon-Khmer speaking Austroasiatics from Malaysia (AAM), Mundari speaking Austroasiatics from Central India (AACI). We also included the Tibeto-Burman population (TB) who were geographically proximal to the Khasis (Figure 1B; Supplementary Figure S1). We considered three Principal Components (PCs) which together could explain 7.1% of the total variation. In the PC1-PC2 space, we found that the three Austroasiatic groups formed distinct clusters. The Khasi did not cluster with either of the other two Austroasiatic populations, instead, clustered with the TB subgroups (Figure 1B). Khasi and TB were distinguished as separate clusters in PC3. Here we could also identify two separate clusters within the TB: one comprising Jamatia and Tripuri (that clustered closer to the AAM) and the other comprising M-Brahmin and Tharu (Supplementary Figure S1), who are known to have been admixed with other populations of North India and the Upper Gangetic plains (Basu, Sarkar-Roy, and Majumder 2016). A similar pattern of clustering was found in the TreeMix analysis (Supplementary Figure S2). The Khasis clustered with the Tibeto Burmans in a branch separate from the other two Austroasiatic populations. The Munda and Mon-Khmer populations also formed distinct clusters.

On the same set of the population (as used in Figure 1B), we surveyed the allele frequencies and calculated pairwise F_st_ (Weir and Cockerham 1984) between them using PLINKv1.9. Here again, we found F_st_ between the Khasi and TB groups to be low (mean = 0.019) (Supplementary Figure S3) which was even lower than those between Khasi and AAM (mean = 0.061) and between Khasi and AAI (mean = 0.033).

In our analysis, *f3* (Mbuti Pygmy; Khasi, X) we used the African Mbuti Pygmy as the outgroup. We measured the *f3* values of Khasi with AAM, AACI, and TB (details in Materials and Methods, Supplementary Table S3). The mean *f3* values are highest (mean = 0.279) between Khasi and TB, indicative of an exclusive and recent shared genetic history. The mean *f3* values were higher (mean = 0.277) for Khasi-AAM than between Khasi-AAI (mean = 0.265). The apparent discordance in the pattern of f3 and F_st_ values when Khasi are compared to AACI and AAM is largely due to the fact that F_st_ is affected by drift. It is to be noted here that we see the AAM populations be highly drifted in our Treemix analyses (Supplementary Figure S2).

**FIGURE 2:**
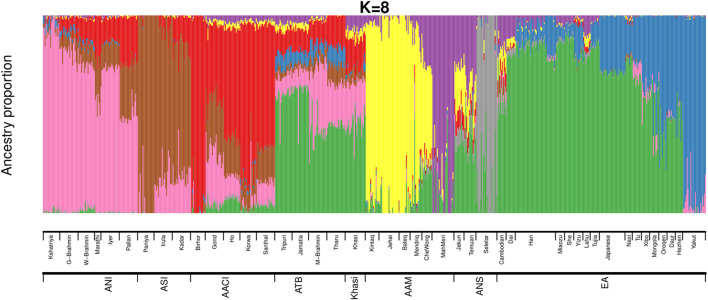
ADMIXTURE analysis on Mainland Indian populations, Malaysians and East Asians of HGDP (ANI: Ancestral North Indian, ASI: Ancestral South Indian, AACI: Austroasiatics of Central India, ATB: Ancestral Tibeto Burman, AAM: Austroasiatics of Malaysia, ANS: Austronesians, EA: East Asians of HGDP).

We then estimated the genomic ancestries and admixture proportions at an individual level by considering all populations from India and Malaysia (the same populations we used in Figure 1A). We also included the East Asians in this analysis because we had observed in our previous study (Tagore et al., 2021) that an East Asian ancestry component was found among some Austroasiatic populations. We did an ADMIXTURE (Alexander, Novembre, and Lange 2009) analysis (Figure 2; Supplementary Figures S4A,B) where the Cross-Validation Error (CVE, details in Materials and Methods) was minimized at *k* = 8 (Supplementary Figure S4A). We also calculated the ancestry proportions for each of these populations (Supplementary Table S3).

The ancestry proportions of the Khasi, as estimated by ADMIXTURE, are distinct from the other two Austroasiatic groups (AAM and AACI) but are very similar to that of the Tibeto Burmans, especially to the Jamatia and Tripuri. The major ancestry identified among the Khasi was also the predominant ancestry identified among the Southern-EA populations (like Dai). In the ADMIXTURE plot (Figure 2), it is depicted by the green color (nearly 44%, “Southern-EA-major” in Supplementary Table S3). This green-colored component is the ancestry modal to the Southern East Asians (henceforth referred to as “Southern EA-like” ancestry) such as Dai. The Khasi genome also has a substantial proportion of AACI-like ancestry (16%, “AACI-major”; red in color) and AAM-like ancestry (4%; yellow color modal to Jehai and 7%; purple color modal to MahMeri). 20% of the Khasi genome is of ANI-like ancestry (“ANI-major”, pink in color). Neither Khasi nor the TB groups had in them any distinctly identified “Khasi-like” or “TB-like” component respectively. Alternatively, the AACI and AAM had genomic components mostly exclusive to them: 64% “AACI-major” component (red in color) and 62% “AAM-major” components (yellow in color) respectively. The East Asian component present in Khasis (green in color) was also present in AACI and AAM, though in lesser proportions of 3.5% and 7.6% respectively. The TBs, on the other hand, had an even higher proportion of this component (51%). In addition to this East Asian component, TBs also have a substantial proportion (7.4%) of a second East Asian component (blue in color). This East Asian component (henceforth referred to as “Northern-EA-major”) is modal in the East Asian populations residing in today’s Northern China (e.g. the Yakut). It is to be noted here that these “Northern-EA” and “Southern-EA” components were also identified in our previous study. Although the TB (particularly Jamatia and Tripuri) and Khasi individuals cluster close in the PCA, this ancestry is negligible in the Khasis. Compared to the Southern EA-like ancestry, the Northern EA-like ancestry is negligible in the other two Austroasiatics groups (AACI and AAM) as well.

**FIGURE 3:**
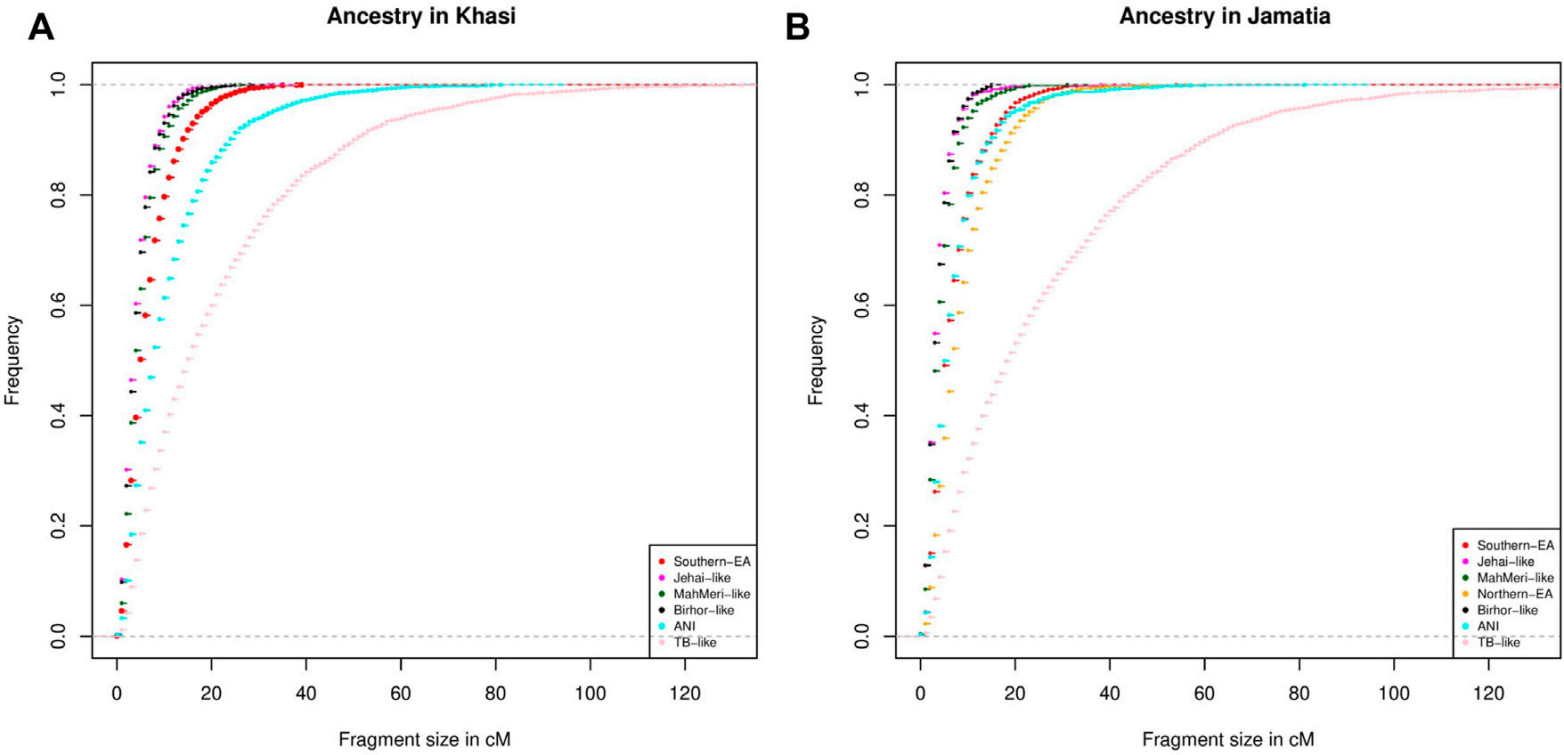
**(A)** Distribution of tract lengths corresponding to different ancestries in Khasi; **(B)** Distribution of tract lengths corresponding to different ancestries in Jamatia.

To investigate the chronology of admixture events into Khasi, we performed local ancestry estimation, using RFMix (Maples et al., 2013). We identified regions within genomes of Khasi individuals representing different ancestries as inferred in the ADMIXTURE analysis. We estimated the length of admixed tracts representing the following ancestries: “Jehai-like”, Birhor-like”, “MahMeri-like”, ANI-like” and “Southern -EA-like”. We looked into the cumulative frequency distribution of these tract lengths. Larger tracts (segments) would correspond to a recent introduction of the corresponding ancestry, and hence can be used as an indicator of the sequence of admixture events. We found that the tract lengths corresponding to Birhor-like and Jehai-like ancestry are the smallest in the Khasis. This is followed by MahMeri-like, ANI-like and Southern EA-like ancestry tracts. This indicates that Southern EA-like ancestry is most recently introduced in the Khasis (Figure 3A).

We then repeated the same analysis with the Jamatia (a subgroup of the TB) and with the same five ancestries i.e. Birhor-like, Jehai-like, MahMeri-like, ANI-like, and Southern EA-like ancestries. Furthermore another ancestry: the Northern EA-like was also included in the analyses because this was an additional ancestry present substantially in the TBs (as evident from ADMIXTURE analysis) but was absent among the Khasis. Similar to what was observed in the Khasis, tract lengths corresponding to the Birhor-like and Jehai-like ancestry are the smallest followed by MahMeri-like, ANI-like, Southern EA-like ancestries. This mimics the chronology of the admixture events that we see in the Khasis. However, the largest length of admixture tracts corresponded to the Northern EA-like ancestry (Figure 3B). This indicates that though the overall sequence of admixture i.e. introduction of ancestries within the Khasis and TBs are similar, the introduction of the Northern EA-like ancestry is the most recent event and unique to the TBs. Thus we conclude that the admixture of the East Asian populations and ancestors of present-day Khasi and Tibeto Burmans is a relatively recent event; of the two distinct East Asian genetic ancestries, the Northern-EA ancestry was introduced in the Tibeto Burmans subsequent to the Southern EA-like ancestry. While both Khasis and TBs have experienced multiple admixture events, the Northern East Asian admixture largely with the TBs is the one which is unique and recent.

We further dated these local admixture events using a method implemented in MOSAIC (Salter-Townshend and Myers 2019) that infers admixture time by fitting an exponential decay coancestry curve (details in Supplementary Material). We chose homogeneous representative populations such as Yakut for Northern EA-like ancestry, Miazou for Southern EA-like ancestry and Birhor for Central Indian Austroasiatic ancestry, as a source population for admixture in populations such as Khasi and Tibeto-Burman. We found that the last evidence of admixture between Southern EA-like ancestry-bearing populations and Austroasiatics and TB took place 13.9 and 10.5 generations ago when Khasi and Jamatia (a representative subgroup for the Tibeto Burman population) were chosen as recipients (Supplementary Figures S5A,B). We also found that the incorporation of Northern EA-like ancestry in the Jamatia happened as recently as 8.3 generations ago (Supplementary Figure S5C). These findings were in accordance with the chronology of events we inferred from the RFMix analysis. This substantiates our conclusion that there were at least two distinct admixture events in NEI populations with East Asians where populations bearing Southern EA-like ancestry admixed first with both the ancestors of TB and Khasi and later populations bearing Northern EA-like ancestry admix mostly with the ancestors of TB.

## Discussion

The Khasi are a relatively large population, subdivided into groups owing to geographical barriers, and show considerable heterogeneity as evident from an anthropometric study (Das 1970). We find from our analyses (PCA, ADMIXTURE, TreeMix), that the Khasis are genetically very similar to the Tibeto-Burmans. The PCA cannot identify the Khasis as a distinct cluster, separate from other TB populations when compared with AACI and AAM. The Khasi Austroasiatics are distinct from the other Austroasiatics (Mundari or AACI and Mon-Khmer or AAM) in our study which conforms to the linguistic classification by Diffloth (2005b). The ancestral components inferred using ADMIXTURE in the Khasis and TBs are also very similar. In our previous study, we had observed that the AACI, TB, and AAM shared a deep common ancestry and proposed that all of their ancestors likely spoke some proto-AA language. The observed genomic profile of the Khasis suggests that the Austroasiatic-speaking Khasis fit well into the proposed model. We had also postulated that the ancestors of the present-day TB and the AAM populations experienced admixture with southward migrating EA agriculturists. Here we find that the Khasis, residing in the same region as the TBs, experienced the same sequence of admixture events as the Jamatia (TB). This indicates they likely share a common history. The southward migration of East Asians led to the incorporation of East-Asian ancestry in the Tibeto Burmans and the Khasis. This migration was extensive and as we have also previously observed, the admixture signals of this migration can also be found among other AA speakers (predominantly the AAM).

Despite an overall genomic similarity of the TBs and Khasis, there is a distinct difference between the TB populations and the Khasis: unlike the TBs, the Khasis lack Northern East Asian ancestry. Results from our RFMix analysis suggest that there were at least two distinct waves of East Asian migration. The first wave brought the Southern East Asian ancestry that got incorporated in both the Khasis and the TBs and the second wave brought the Northern East Asian component. This migration started possibly from Northern EA and led to the introduction of the Northern EA-like genomic ancestry into the TB population but not in the Khasis. It is to be noted that the Northern EA component is absent from other Austroasiatic speakers as well (AACI and AAM), although some of these populations have substantial EA ancestry, i.e Southern EA ancestry. Wang et al. (2018) suggested that the TBs were an admixed group resulting from two distinct ancient populations: a hunter-gatherer population, (which we believe were the proto-Austroasiatics) and a millet farmer population from middle Yellow River basin. A genetic link between the millet farming proto-Sino Tibetans of the Yellow River basin and Tibeto Burmans has also been proposed by Guo et al. (2022). Though Wang et al propose a two wave migration leading to the formation of TBs, they propose that out of the two, only one wave of migration formed both the TBs of India and populations of MSEA. However, our study suggests that though there were atleast two waves of migration, the second wave solely affected the TBs while the first affected both the TBs and the AAs of MSEA.

We, therefore, propose, in agreement with our previous study, that the ancestors of extant Austroasiatic speakers were widespread across Central India and Southeast Asia encompassing the present-day location of the TBs and Khasis. This is in agreement with other studies (Cordaux et al., 2004). It is hence plausible that the ancestors of present-day Tibeto-Burman speakers spoke some form of an Austroasiatic or Proto-Austroasiatic language. With time, the Austroasiatic populations evolved into three major branches as we see them today namely Mundari, Mon-Khmer, and Khasi.

Higham has suggested that before Neolithic expansion, this region was inhabited by hunter gatherers (Higham 2017). He also suggested that the expansion of farming communities happened from two regions that reached mainland Southeast Asia: one of millet cultivators from the Yellow River basin and another of the rice cultivators from the Yangtze River basin. Such migration events are also supported by morphological studies. Cranial (Matsmura 2011) and Dental (Matsmura 2010) morphological studies found two groups of individuals at the Man Bac excavation site in Southeast Asia: one close to the Neolithic inhabitants of Weidun in the Yangtze Valley and the other to the local hunter gatherers. Archaeological studies in Southeast Asia also supports presence of hunter-gatherers in Southeast Asia as well as Southern China (Higham 2013) and that archaeological sites provide indication that immigrants from Southern China encountered these hunter gatherers on their way. Infact, Neolithic migration has also diluted the genetic differentiation within China (Yang et al., 2020). An extensive documentation of rice spread also supports the spread of rice from China to Southeast Asia (Fuller et al., 2010).

We argue that the language of the extant TBs is a result of this linguistic shift, possibly evidence of elite dominance, which is a consequence of the migration and gene flow from Northern East Asia. When we look at the ancestral segments of Northern-EA ancestry, we find that they are among the longest ancestral segments in TB, preceded by segments of Southern EA-like ancestry. We postulate that the two migration events from East Asia were such that initially, populations bearing Southern EA-like ancestry arrived in NEI, and later came the populations of Northern EA-like ancestry. The Southern EA-like ancestral segments are also present in Khasis and AAM, the two Austroasiatic groups with substantial East Asian ancestry. In these populations, Southern EA-like ancestral segments are among the longest. The AACI however have negligible East Asian components in their genome. It is to be noted here that the language of the AACI, i.e. Mundari is much more distant from the other two branches of the AA family, namely Khasi-Khmuic and Mon-Khmer. The Khasi-Khmuic and Mon-Khmer are more similar to the Sino-Tibetan language. This is expected as our genetic data also confirms closer proximity and longer admixture of Khasi-Khmuic and Mon-Khmer speaking populations (Khasi and AAM) with Southern-EA populations. The admixture with populations of Northern EA-like ancestry is unique among the TB and their languages belong to Sino-Tibetan, a different language family altogether. In TBs this ancestry has been incorporated after the second migration wave. This leads us to conclude that the ancestral populations of TB have experienced a language shift, from a more proto-Khasi-Khmuic language to a language closer to that of the East Asians (the Tibeto Burman languages) and this has occurred due to the most recent admixture with populations with Northern EA-like ancestry.

## Data Availability

The datasets presented in this study can be found in online repositories. The names of the repository/repositories and accession number(s) can be found below: https://share.nibmg.ac.in/d/0b373e011a1d4f689cb2/, BMCB2021; https://www.nibmg.ac.in/fp/khasi_data.html, Khasi_data_2022.
