# Supplementary figures and images for: Multiple migrations from East Asia led to linguistic transformation in NorthEast India and mainland Southeast Asia
Source: Front Genet. 2022 Oct 11;13:1023870. doi: 10.3389/fgene.2022.1023870 (PMC9592996; doi:10.3389/fgene.2022.1023870)

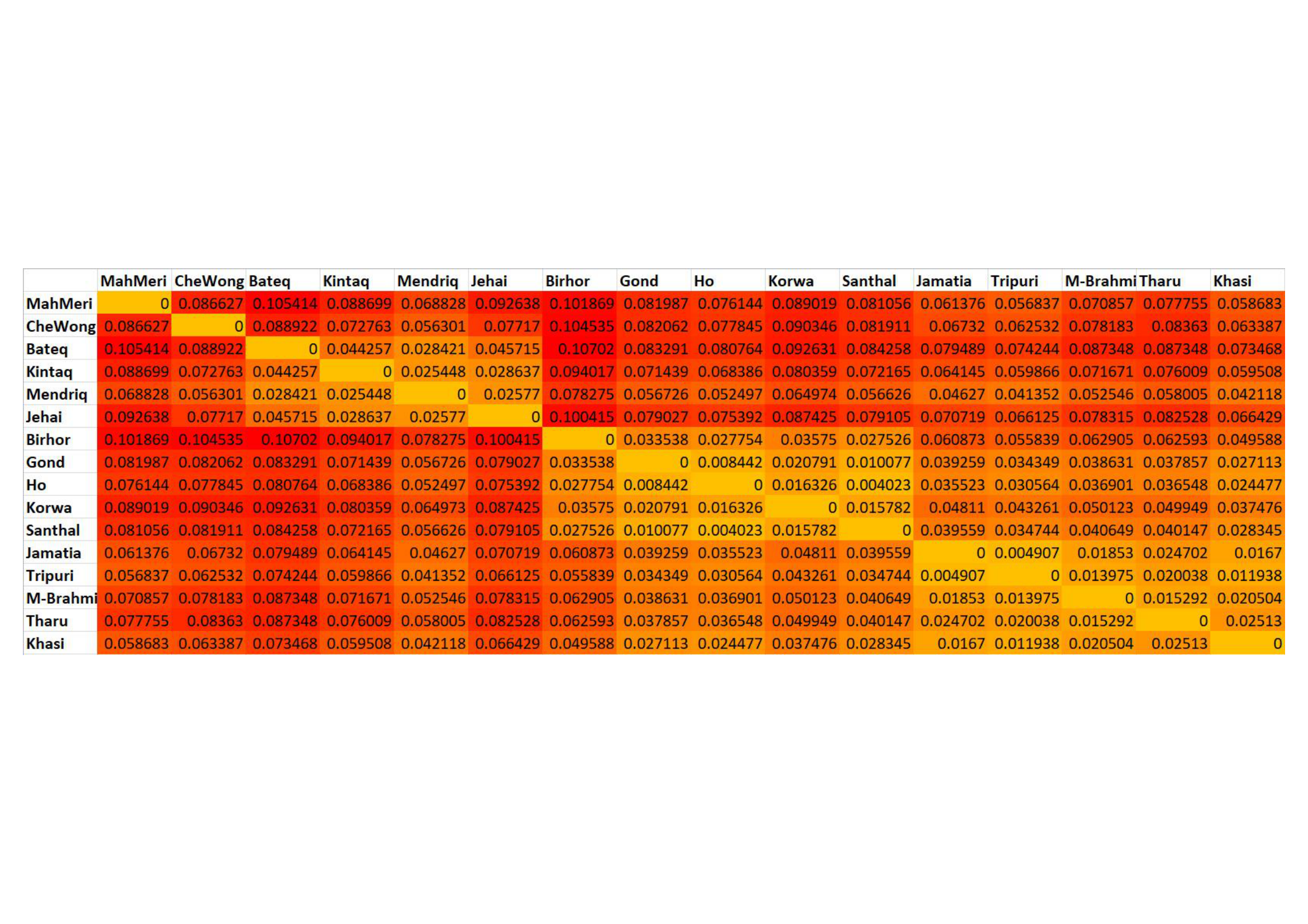

Supplement: Supplementary file 1 [file Image3.TIFF]

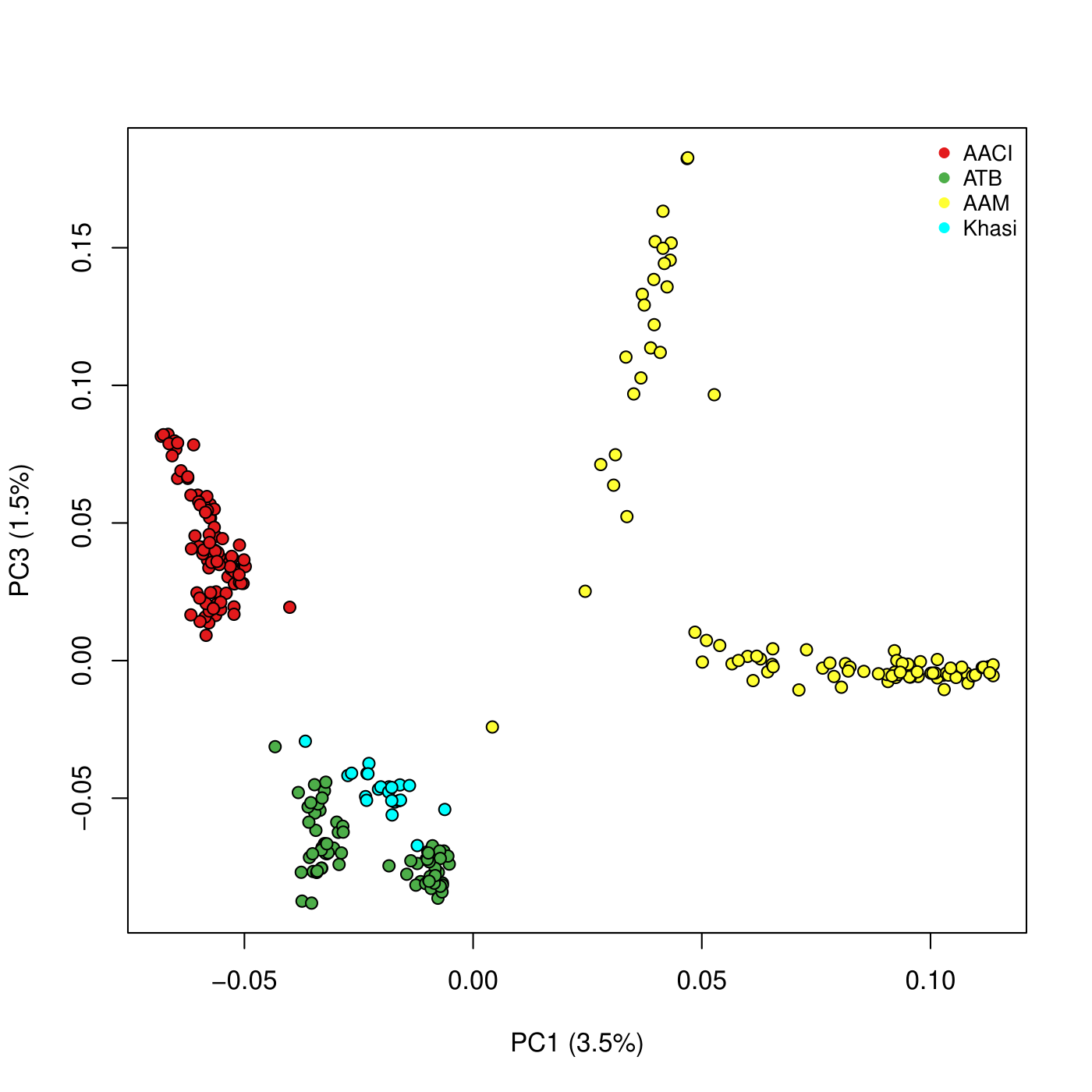

Supplement: Supplementary file 2 [file Image1.TIFF]

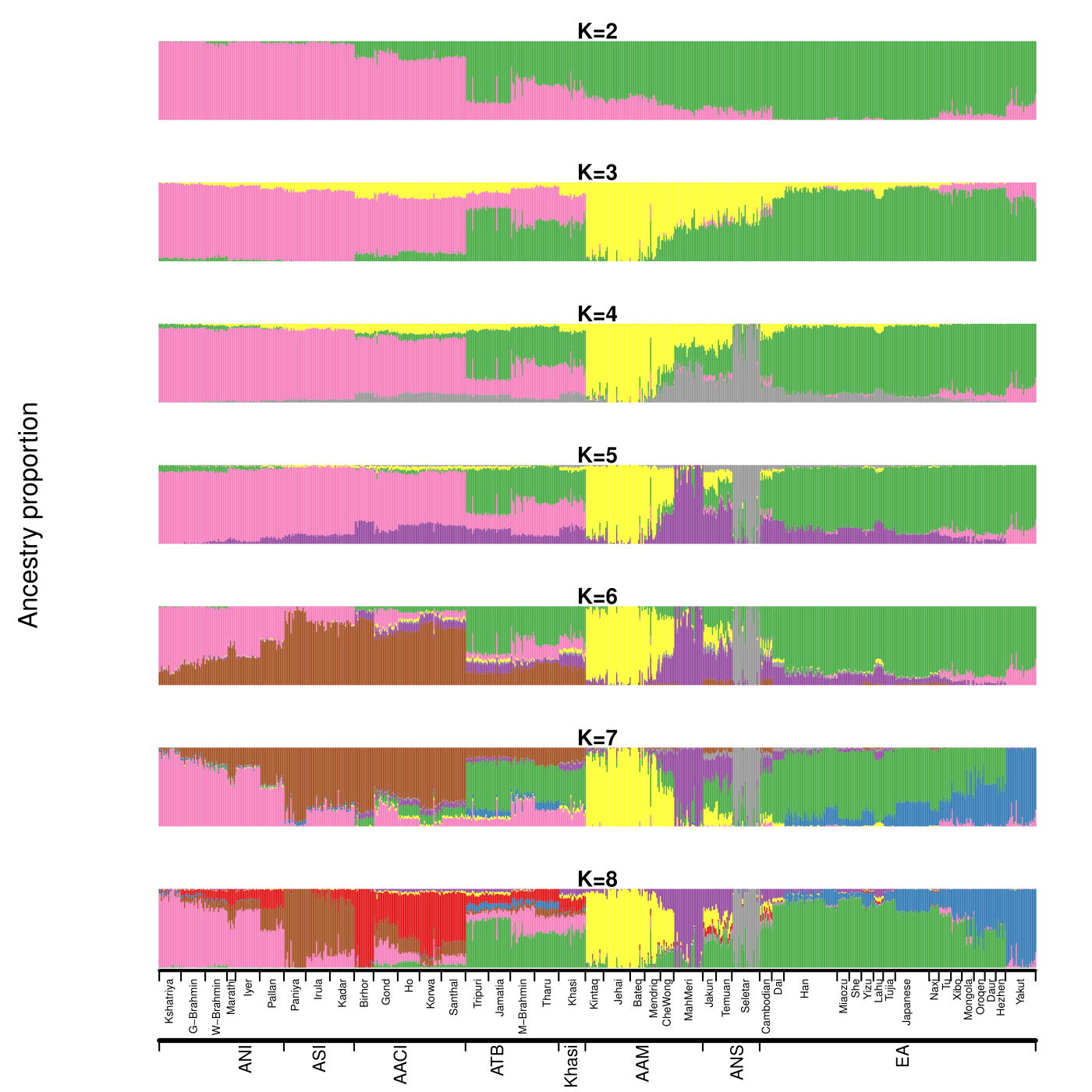

Supplement: Supplementary file 3 [file Image5.TIFF]

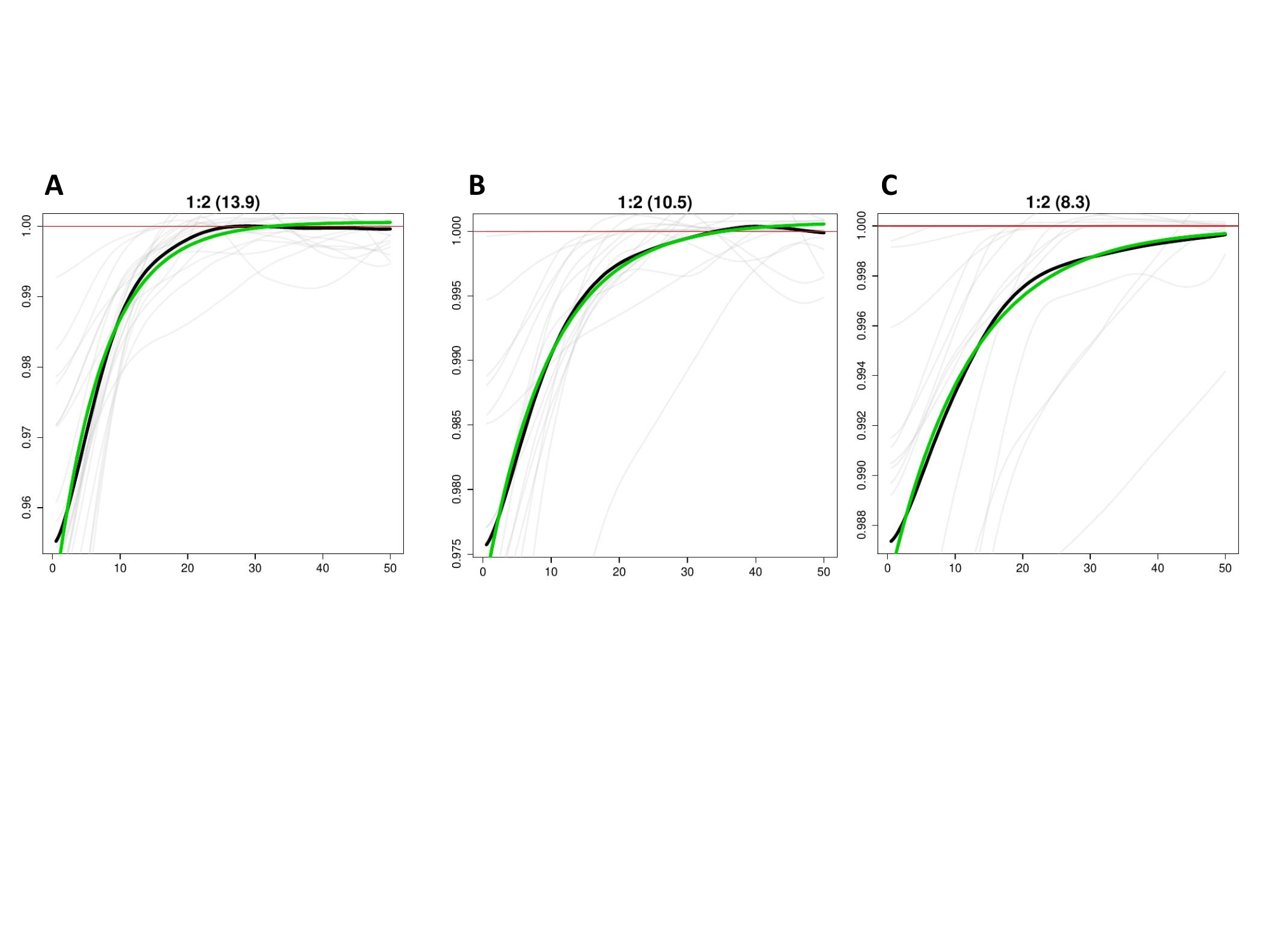

Supplement: Supplementary file 4 [file Image6.TIFF]
