# Supplementary material for: Multiple migrations from East Asia led to linguistic transformation in NorthEast India and mainland Southeast Asia
Source: Front Genet. 2022 Oct 11;13:1023870. doi: 10.3389/fgene.2022.1023870 (PMC9592996; doi:10.3389/fgene.2022.1023870)
Supplement: Supplementary file 7 [file DataSheet1.docx]

**Supplemental Data**

**Figure S1**: **Principal Components (PC) Analysis with Austroasiatics and Tibeto Burmans**: Plot of PC1 vs PC3

**Figure S2**: **TreeMix Analysis with Austroasiatics and Tibeto Burmans**: Maximum likelihood tree generated by Treemix on the subpopulations of AACI, TB, AAM and Khasi with Mbuti pygmies as outgroup showing population separation and drift.

**Figure S3**: **Fst estimation**: Heatmap generated on the estimated weighted Fst values between subpopulations belonging to mainland Indian and Malaysian populations. The different subgroups are labelled on the right and the bottom of the heatmap. Light yellow colour represents low Fst value while dark red represents high Fst value.

**Figure S4A: ADMIXTURE analysis on populations from mainland India, Malaysia, East Asians (EA) of HGDP:** Cross-Validation Error graph

**Figure S4B: ADMIXTURE analysis on populations from mainland India, Malaysia, East Asians (EA) of HGDP:** ADMIXTURE cluster graph

**Figure S5:** **Admixture dating using coancestry curves.** Y axis is the relative probability of jointly copying two chunks from the donor populations and the x axis is the genetic distance in centimorgans.  The green line represents the fitted curve, the black line represents the across targets observed ratios, and the grey lines represent the per target ratio. At the top of each panel is the index of the pair of ancestries being examined as a:b (where the a and b represent the indices of the ancestries). The adjacent number inside parenthesis represents the number of generations since admixture. For the different panels a:b represent (A) Birhor:Maizou and Khasi as an admixed group, (B) Birhor:Miazou and Jamatia as an admixed group and (C) Birhor:Yakut and Jamatia as an admixed group

**Table S1A:** The different populations broadly classified, the dataset from which these populations have been obtained and the number of individuals. The abbreviations for some of the names are enclosed within brackets

| **Dataset** | **Populations and their abbreviations** | **Number of individuals** |
| --- | --- | --- |
| HGDP | Africa | 101 |
|  | America | 64 |
|  | Central South Asia (CSA) | 200 |
|  | East Asia (EA) | 228 |
|  | Europe (EUR) | 157 |
|  | Middle East (ME) | 163 |
|  | Oceania (OCE) | 27 |
| Indian populations | Ancestry North India(ANI) | 104 |
|  | Ancestry South India (ASI) | 58 |
|  | Tibeto Burman (TB or ATB) | 77 |
|  | Austroasiatics of Central India(AACI) | 92 |
|  | Island | 36 |
|  | Khasi | 22 |
| Malaysian population | Malaysian Austroasiatics(AAM) | 97 |
|  | Austronesians (ANS) | 47 |
|  | **TOTAL** | **1473** |

**Table S1B:** Populations from India and their corresponding genetic ancestry and linguistic groups. The abbreviations for the names are enclosed within brackets

| **Ancestral group** | **Population subgroups** | **Linguistic group** |
| --- | --- | --- |
| Ancestral North Indian  (ANI) | Kshatriya (K or KSH) | Indo-European |
|  | Gujrati Brahmins (GBR) |  |
|  | West Bengal Brahmins (BR2) |  |
|  | Marathi (MT) |  |
|  | Iyer (IR) | Dravidian |
|  | Pallan (PLN) |  |
| Ancestral South Indian  (ASI) | Kadar (KA) |  |
|  | Irula(IL) |  |
|  | Paniya(PY) |  |
| Ancestral Tibeto Burman  (ATB or TB) | Manipuri Brahmin (MPB) | Tibeto Burman |
|  | Jamatia (JAM) |  |
|  | Tripuri (TRI) |  |
|  | Tharu(TH) | Indo-European |
| Ancestral Austroasiatic (AAA) | Bihor (BIR) | Austroasiatic  (Munda/Mundari) |
|  | Gond (GD) |  |
|  | Ho (HO) |  |
|  | Korwa(KO) |  |
|  | Santhal (SA) |  |
| Island ancestry | Jarwa(JW) | Unclassified |
|  | Onge (ONG) |  |
| Unclassified | Khasi | Austroasiatic  (Khasi-Khmuic) |

**Table S1C:** Populations from Malaysia and their corresponding linguistic group. The abbreviations for the names are enclosed within brackets

| **Population subgroups** | **Linguistic group** |
| --- | --- |
| Bateq (BTQ) | Austroasiatic (AAM) |
| CheWong (CW) |  |
| Jehai (Jeh) |  |
| Kintaq (Kin) |  |
| Mendriq (Men) |  |
| MahMeri (Mah) |  |
| Jakun(JKN) | Austronesian (ANS) |
| Temuan(T) |  |
| Seletar(S) |  |

**Table S2:**  **Values of outgroup f3(Mbuti Pygmy; X,Y) statistics.** X is Khasi and Y is AACI, AAM and TB subgroups

| X | Y | f3 | Z |
| --- | --- | --- | --- |
| Khasi | Birhor | 0.265851 | 121.538 |
| Khasi | Gond | 0.261069 | 122.326 |
| Khasi | Ho | 0.266841 | 123.2 |
| Khasi | Korwa | 0.266538 | 123.834 |
| Khasi | Santhal | 0.26609 | 123.431 |
| Birhor | Bateq | 0.263538 | 116.809 |
| Birhor | Mendriq | 0.26644 | 119.175 |
| Birhor | Jehai | 0.265502 | 117.42 |
| Birhor | Kintaq | 0.264335 | 117.258 |
| Birhor | CheWong | 0.267447 | 118.545 |
| Birhor | MahMeri | 0.267884 | 117.445 |
| Gond | Bateq | 0.257851 | 118.492 |
| Gond | Mendriq | 0.259895 | 119.693 |
| Gond | Jehai | 0.259155 | 118.165 |
| Gond | Kintaq | 0.258423 | 118.108 |
| Gond | CheWong | 0.260691 | 119.814 |
| Gond | MahMeri | 0.260565 | 117.51 |
| Ho | Bateq | 0.26441 | 118.153 |
| Ho | Mendriq | 0.267181 | 119.924 |
| Ho | Jehai | 0.266118 | 118.266 |
| Ho | Kintaq | 0.265071 | 118.57 |
| Ho | CheWong | 0.268449 | 119.67 |
| Ho | MahMeri | 0.269004 | 118.789 |
| Korwa | Bateq | 0.265046 | 118.752 |
| Korwa | Mendriq | 0.2674 | 120.385 |
| Korwa | Jehai | 0.266471 | 119.102 |
| Korwa | Kintaq | 0.265566 | 119.362 |
| Korwa | CheWong | 0.268697 | 120.315 |
| Korwa | MahMeri | 0.26878 | 118.232 |
| Santhal | Bateq | 0.263551 | 118.193 |
| Santhal | Mendriq | 0.26612 | 119.853 |
| Santhal | Jehai | 0.265157 | 118.11 |
| Santhal | Kintaq | 0.264112 | 118.266 |
| Santhal | CheWong | 0.267196 | 119.484 |
| Santhal | MahMeri | 0.267227 | 118.282 |
| Khasi | Jamatia | 0.283278 | 124.3 |
| Khasi | Tripuri | 0.28337 | 124.59 |
| Khasi | M-Brahmin | 0.272437 | 124.249 |
| Khasi | Tharu | 0.275304 | 123.501 |

**Table S3: Ancestry proportion estimation:** Estimation of population cluster ancestry proportion based on ADMIXTURE analysis on Mainland Indian, Malaysian, HGDP-East Asians

|  | Southern-EA–major (green) | Seletar- major (grey) | ANI- major (pink) | ASI- major (brown) | Nortthern-EA– major (blue) | AACI- major (red) | AAM- major (yellow) | MahMeri- major (purple) |
| --- | --- | --- | --- | --- | --- | --- | --- | --- |
| ANI | 0.557 | 0.452 | 71.994 | 12.526 | 1.459 | 11.973 | 0.401 | 0.638 |
| ASI | 0.011 | 0.086 | 13.776 | 79.027 | 0.066 | 6.721 | 0.224 | 0.088 |
| AACI | 3.471 | 0.779 | 10.966 | 16.147 | 0.453 | 63.883 | 1.759 | 2.543 |
| TB | 50.967 | 0.327 | 18.991 | 5.616 | 7.436 | 12.505 | 1.802 | 2.356 |
| Khasi | 44.227 | 1.607 | 20.487 | 4.856 | 1.484 | 16.042 | 4.135 | 7.163 |
| AAM | 7.578 | 1.182 | 0.331 | 0.228 | 0.307 | 0.824 | 62.418 | 27.131 |
| Austronesian | 24.899 | 44.701 | 0.452 | 0.068 | 0.271 | 3.871 | 9.498 | 16.240 |
| EA | 63.719 | 0.914 | 1.774 | 0.159 | 29.039 | 0.631 | 0.950 | 2.813 |
